# *Salmonella enterica* *serovar* *Enteritidis* brain abscess mimicking meningitis after surgery for glioblastoma multiforme: a case report and review of the literature

**DOI:** 10.1186/s13256-016-0973-9

**Published:** 2016-07-07

**Authors:** Léa Luciani, Grégory Dubourg, Thomas Graillon, Estelle Honnorat, Hubert Lepidi, Michel Drancourt, Piseth Seng, Andreas Stein

**Affiliations:** 1 Aix Marseille Université, URMITE, UM63, CNRS 7278, IRD 198, Inserm 1095, 13005 Marseille, France; 2 Pôle de Maladies Infectieuses, Hôpital de la Timone, Assistance Publique Hôpitaux de Marseille, Institut Hospitalo-Universitaire Méditerranée Infection, 13005 Marseille, France; 3 Service de neurochirurgie, Hôpital de la Timone, Assistance Publique Hôpitaux de Marseille, 13005 Marseille, France; 4 Service des Maladies Infectieuses, Hôpital de la Conception, 147, boulevard Baille, 13005 Marseille, France; 5 Unité de Recherche sur les Maladies Infectieuses et Tropicales Emergentes, Faculté de Médecine, Aix Marseille Université, 27, Boulevard Jean Moulin, 13385 Marseille, Cedex 5 France

**Keywords:** Brain abscess, Glioblastoma, Post-surgery meningitis, *Salmonella*, *Salmonella enterica*, MALDI-TOF, Bacteria, Infection, Human

## Abstract

**Background:**

*Salmonella* brain abscess associated with brain tumor is rare. Only 11 cases have been reported to date. Here we report a case of brain abscess caused by *Salmonella enterica* *serovar* *Enteritidis* mimicking post-surgical meningitis in a patient with glioblastoma multiforme.

**Case presentation:**

A 60-year-old Algerian woman was admitted through an emergency department for a 4-day history of headache, nausea and vomiting, and behavioral disorders. Surgery for cerebral tumor excision was performed and histopathological analysis revealed glioblastoma multiforme. On the seventh day post-surgery, she presented a sudden neurological deterioration with a meningeal syndrome, confusion, and fever of 39.8°C. Her cerebrospinal fluid sample and blood cultures were positive for *S. enterica Enteritidis.* She was treated with ceftriaxone and ciprofloxacin. On the 17th day post-surgery, she presented a new neurological disorder and purulent discharge from the surgical wound. Brain computed tomography revealed a large cerebral abscess located at the operative site. Surgical drainage of the abscess was performed and microbial cultures of surgical deep samples were positive for the same *S. enterica Enteritidis* isolate. She recovered and was discharged 6 weeks after admission.

**Conclusions:**

In this case report, a brain abscess was initially diagnosed as *Salmonella* post-surgical meningitis before the imaging diagnosis of the brain abscess. The diagnosis of brain abscess should be considered in all cases of non-typhoidal *Salmonella* meningitis after surgery for brain tumor. Surgical brain abscess drainage followed by prolonged antibiotic treatment remains a major therapeutic option.

## Background

*Salmonella* species are mainly known as common agents of gastroenteritis worldwide. Invasive *Salmonella* infections have been reported due to their potential to cause focal suppurative complications in urinary tract infection, osteoarticular infection and liver abscess [1]. Central nervous system *Salmonella* infection is rare and occurs primarily in young children [2] and immunocompromised adults, including human immunodeficiency virus (HIV) infection and co-infected patients [3] and chronic granulomatous disease [4]. Here, we report a case of brain abscess caused by *S. enterica* subspecies (subsp.) *enterica* *serovar Enteritidis* mimicking post-surgical meningitis in a patient with glioblastoma multiforme. We also review cases of *Salmonella* brain abscess in patients with cerebral tumors.

## Case presentation

**Fig. 1:**
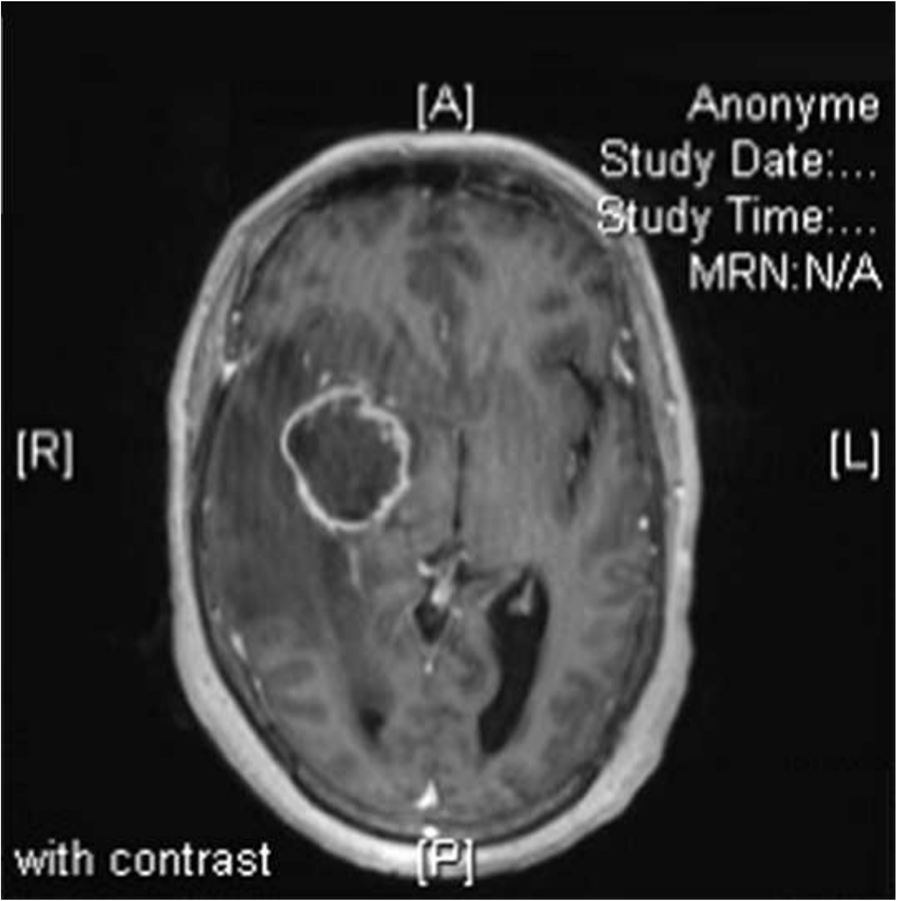
Brain magnetic resonance imaging revealed a single 40×35 mm tumor in the right mesial temporal region and a mass effect compression of the right lateral ventricle with transtentorial herniation

**Fig. 2:**
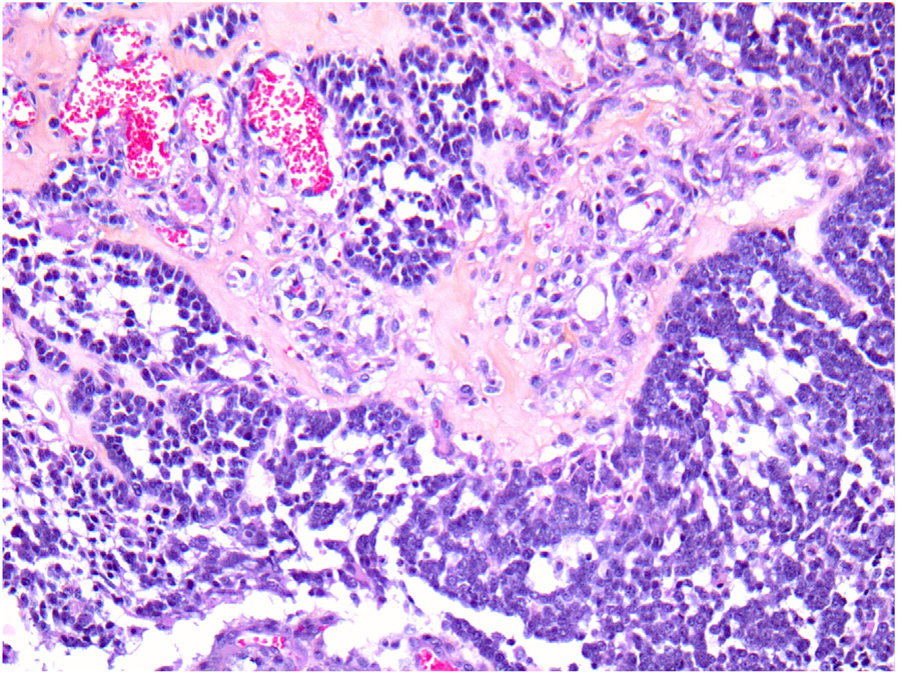
A histological examination revealed glioblastoma multiforme without any microorganism identified on histological analysis

In September 2015, a 60-year-old Algerian woman was seen in the emergency department in Marseille, France for a 4-day history of headache, nausea and vomiting, and behavioral disorders. She had an unremarkable medical history apart from obesity (body mass index at 30.9 kg/m^2^). Brain magnetic resonance imaging (MRI) revealed a single 40×35 mm tumor in her right mesial temporal region and a mass effect compression of her right lateral ventricle with transtentorial herniation (Fig. 1). She was transferred to our neurosurgery department, where levetiracetam and methylprednisolone led to neurological improvement. At that time, her leukocyte count was elevated at 22×10^9^/L (neutrophil count was 21×10^9^/L, lymphocytes were decreased at 0.47×10^9^/L, and her platelet count was 291×10^9^/L). Surgery for tumor removal was performed on day 5 of her admission. A histological examination revealed glioblastoma multiforme (Fig. 2). No bacteria were seen on histological analysis.

On the seventh day post-surgery, she presented a sudden neurological deterioration with a meningeal syndrome, confusion and fever of 39.8 °C. Laboratory investigations revealed an elevated leukocyte count at 13×10^9^/L, elevated neutrophils at 12.62×10^9^/L, low lymphocytes at 0.15×10^9^/L, normal platelets at 154×10^9^/L, and elevated C-reactive protein at 304 mg/L. Cerebrospinal fluid (CSF) sample analysis revealed an elevated protein level of 2.93 g/L, a low glucose level of 0.1 mmol/L, and a leukocyte count of 5400 cells/mm3 with 80 % neutrophils. CSF cultures and blood cultures were positive for *S. enterica*. The isolates from the CSF and blood were further identified as *S. enterica* subsp. *enterica* serotype *Enteritidis* as identified by our national reference center for *Salmonella* (Institut Pasteur, Paris). The isolates were susceptible *in vitro* to amoxicillin, ceftriaxone, imipenem/cilastatin, gentamycin, co-trimoxazole and fluoroquinolone.

**Fig. 3:**
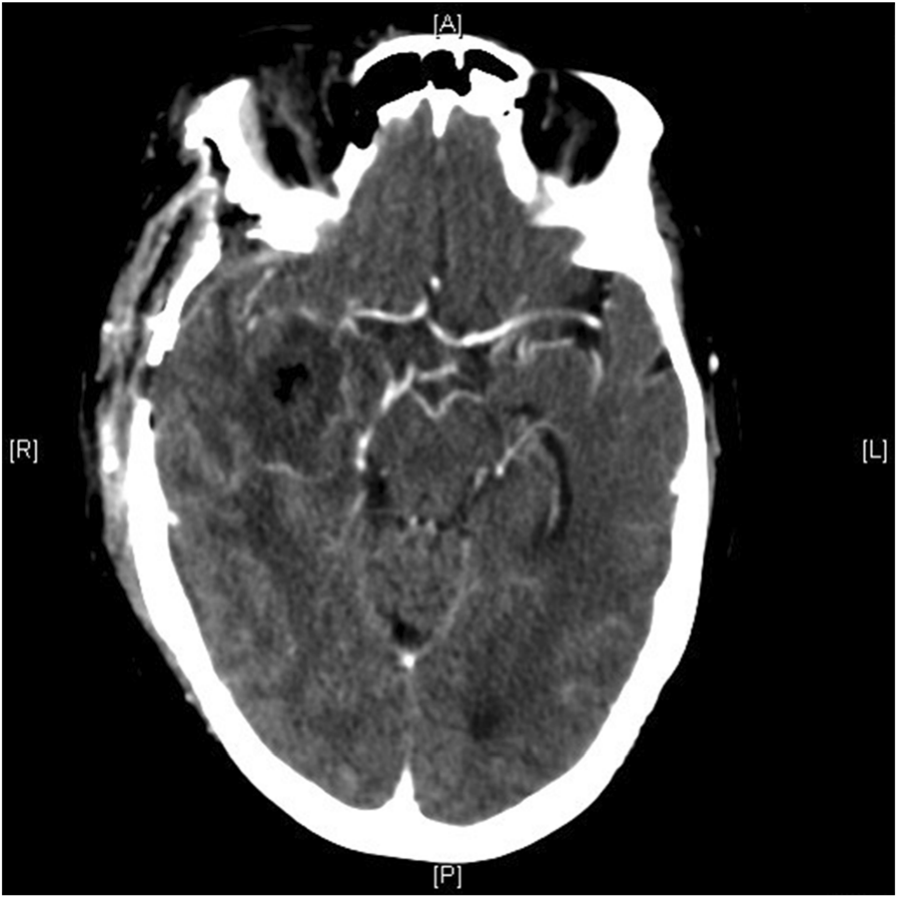
Computed tomography reveals a large cerebral abscess located at the operative site

A diagnosis of *Salmonella* meningitis was made and she was treated with ceftriaxone administered intravenously 2 g/day and oral ciprofloxacin 500 mg every 8 hours. On the 17th day post-surgery, she presented a new neurological disorder and purulent discharge from the surgical wound. Brain computed tomography (CT) revealed a large cerebral abscess located at the operative site (Fig. 3). Surgical drainage of the abscess was performed by craniotomy, which confirmed the diagnosis of intraparenchymal abscess located at the glioblastoma resection site. Microbial cultures of surgical deep samples were positive for *S. enterica* subsp. *enterica* *serovar Enteritidis*, which were susceptible to all antibiotics tested above. She was discharged 6 weeks after admission. Prolonged 10-day anaerobic bacterial cultures of her CSF, bloodstream and brain abscess were negative. A combination of ceftriaxone-ciprofloxacin was given for 6 weeks, and ciprofloxacin treatment was prolonged for 3 months because of the infectious risk due to chemotherapy immunosuppression. No neurological sequelae were noted. Evaluation of the immune system remained normal and HIV serology was negative.

## Discussion

Here we report a case of brain abscess due to *S. enterica* subsp*. enterica* *serovar Enteritidis* mimicking meningitis occurring after surgery for glioblastoma. *Salmonella* brain abscesses are rarely reported. Only a few cases of typhoidal *Salmonella* brain abscess have been reported in immunocompetent adults, usually related to situations promoting their incidence, including recent travel in endemic areas [5], typhoid fever [6], or ingestion of contaminated milk [7]. To the best of our knowledge, only 11 cases of *Salmonella* brain abscess associated with brain tumor have been reported [8–18]. Most of these cases (nine cases) were caused by non-typhoidal *Salmonella,* including eight cases of *S. enterica* Enteritidis and one case of *Salmonella enterica* Typhimurium. However, *S. enterica* Typhimurium is usually responsible for invasive human salmonellosis [19]. Glioblastoma is the main type of brain tumor that has been associated with *Salmonella* brain abscess (four cases), and all of these cases were caused by *S. enterica* Enteritidis (Table 1).

**Table 1:** Review of 12 cases of *Salmonella* brain abscess related to brain tumors reported in the literature and in our case

Cases	Age, sex, geographical origin	Cerebral tumor	Tumor surgery before diagnosis of brain abscess	Patients under systemic corticosteroid treatment	Clinical symptoms	Surgical drainage	Antibiotic treatment	*Salmonella* species	Clinical outcome
Our case (2015)	60 years, female, Algeria	Yes, multiforme glioblastoma	Yes	Yes	Sudden neurological deterioration, meningeal syndrome	Yes	Yes, 3 months	*Salmonella Enteritidis* (CSF, blood, pus, brain abscess)	Good
Rodriguez, Valero, and Watanakunakorn 1986 [8]	28 years, male, Ohio (USA)	Yes, metastatic carcinoma	No	Yes	Per orbital pain, nausea, papilledema	Yes	Yes, 6 weeks (radiotherapy)	*Salmonella Enteritidis* (brain tissue and blood)	Good
Sharma, Raja, and Shivananda 1986 [9]	32 years, male, India	Yes, malignant astrocytoma	Yes	No	Headache, vomiting, somnolence	Yes	Yes, unknown duration	*Salmonella Typhi*	Good
Noguerado *et al.* 1987 [10]	78 years, male, Spain	Yes, multiforme glioblastoma	No	Yes	General conditions deteriorated, fever, meningeal syndrome, septic shock	No	Yes	*Salmonella Enteritidis* (CSF and blood)	Died
Bossi *et al.* 1993 [11]	24 years, male, Tunisia	Yes, multiforme glioblastoma	Yes	Yes	Fever, confusion	Yes	Yes, unknown duration	*Salmonella Enteritidis* (CSF, blood and brain abscess)	Good
Shanley and Holmes 1994 [12]	28 years, female, Hawaii (USA)	Yes, craniopharyngioma	No	Not mentioned	Sudden loss of vision	Yes, Hypophysectomy to decompress optic chiasm	Not mentioned	*Salmonella Typhi* (pus, brain abscess)	Good
Fiteni *et al.* 1995 [13]	49 years, female, France	Yes, astrocytoma	Yes	Yes	Fever, confusion	Yes	Yes, 9 weeks	*Salmonella Enteritidis* (CSF, blood and brain abscess)	Residual hemiparesis
Sarria, Vidal, and Kimbrough Iii 2000 [14]	58 years, female, Texas (USA)	Yes, multiforme glioblastoma	No	Yes	Fever, meningeal syndrome, hemiparesis, coma	Yes	Yes, 6 weeks and local application	*Salmonella Enteritidis* (material)	Died
Kumari and Kan 2000 [15]	59 years, male, Washington (USA)	Yes, metastatic adenocarcinoma	Yes	Yes	Fever, tachycardia, confusion	Yes	Yes, 6 weeks	*Salmonella typhimurium* (cerebral abscess)	Good
Schröder *et al.* 2003 [16]	46 years, female, Germany	Yes, craniopharyngioma	Yes	Yes	Tension, headache at craniotomy site	Yes	Yes, duration not known	*Salmonella Enteritidis* (pus, brain abscess)	Coxitis abscess
Aissaoui *et al.* 2006 [17]	72 years, male, Morocco	Yes, oligodendroglioma	Yes	Yes	Fever, neurological deterioration	No	Yes, 8 days then patient died	*Salmonella Enteritidis* (CSF and blood)	Died
Sait *et al.* 2011 [18]	57 years, male, not known	Yes, multiforme glioblastoma	Yes	No	Headache, discharge wound, meningeal signs	Yes	Yes, 4 weeks	*Salmonella Enteritidis* (material and blood)	Good

*CSF* cerebrospinal fluid

Symptoms of *Salmonella* brain abscess associated with brain tumor are heterogeneous. Most cases (six cases) have occurred after surgical resection of a brain tumor, initially indicated by fever or neurological deterioration and confusion. However, meningeal signs were noted in three reported cases. In our case, the brain abscess was initially diagnosed as *Salmonella* post-surgical meningitis before imaging diagnosis of the brain abscess. In our case, the diagnosis of glioblastoma multiforme was suggested by brain MRI and confirmed by a histological examination of the surgical biopsy. *In vivo* imaging technology, such as molecular imaging, is useful in the diagnosis of brain tumors [20] and might be helpful to differentiate bacterial abscess from tumoral tissues and underlying primary disease [21].

In the literature, *Salmonella* species have been identified in purulent exudates from brain abscesses (six cases) and in blood cultures (six cases) and CSF cultures (four cases). In our case, *Salmonella* isolates were identified in the blood, CSF and brain abscess. Most cases in the literature were treated with systemic corticosteroids for brain tumor (eight cases) when the *Salmonella* brain abscess was diagnosed. The prognosis is relatively good with antibiotic treatment. There is no comparative study on the use of dual antibiotic therapy rather than single antibiotic for this indication. Nevertheless, we decided to treat our case initially with a 6-week combination of ceftriaxone-ciprofloxacin due to a significant risk of immunosuppression related to treatment of the glioblastoma multiforme and the large brain abscess. The duration of antibiotic treatment in the literature varied from 4 weeks to 3 months. Most cases in the literature (nine cases) were treated surgically for the brain abscess. However, three patients died and two patients had complications, including residual hemiparesis in one case and a hip abscess in one case.

Chronic carriage of *Salmonella*, primarily biliary, may persist after infection (about 1 % of cases) [22]. In our case, septic signs and digestive symptoms such as gastroenteritis were absent on admission and the clinical symptoms of brain abscess such as fever, meningeal signs, and neurological deterioration occurred only at 1 week post-surgery for glioblastoma. These phenomena might be explained by *Salmonella*’s tropism for necrotic tissue [23], and the central nervous system infection could be secondary to blood dissemination of *Salmonella* from digestive reservoirs in the bile or intestine. Unfortunately, this hypothesis is difficult to confirm due to the transitory carriage and because a stool culture had unfortunately not been performed.

## Conclusions

*Salmonella* brain abscess is rare but can occur in apparently immunocompetent adult patients with brain tumor. The diagnosis of brain abscess should be considered in all cases of non-typhoid *Salmonella* meningitis after surgery for brain tumor. Prolonged antibiotic treatment after surgical brain abscess drainage remains a major therapeutic option.

## References

[1] Wilkins EGL, Roberts C. Extraintestinal salmonellosis. Epidemiol Infect. 1988;100:361–8. 10.1017/s095026880006711x PMC2249349 3378582

[2] Murphy D, Oshin F. Reptile-associated salmonellosis in children aged under 5 years in South West England. Arch Dis Child. 2015;100:364–5. 10.1136/archdischild-2014-306134 25538189

[3] Aliaga L, Mediavilla JD, López de la Osa A, Melander E, López-Gómez M, de Cueto M, et al. Nontyphoidal Salmonella Intracranial Infections in HIV-Infected Patients. Clin Infect Dis. 1997;25:1118–20. 10.1086/516101 9402368

[4] Ma J-S, Chen P-Y, Lau Y-J, Chi C-S. Brain abscess caused by Salmonella enterica subspecies houtenae in a patient with chronic granulomatous disease. J Microbiol Immunol Infect Wei Mian Yu Gan Ran Za Zhi. 2003;36:282–4. 14723260

[5] Bonvin P, Ejlertsen T, Dons-Jensen H. Brain Abscess Caused by Salmonella enteritidis in an Immunocompetent Adult Patient: Successful Treatment with Cefotaxime and Ciprofloxacin. Scand J Infect Dis. 1998;30:632–3. 10.1080/00365549850161313 10225404

[6] Suzuki Y, Sugiyama Y, Ishii R, Sato I. Brain abscess caused by Salmonella typhi. J Neurosurg. 1976;45:709–11. 10.3171/jns.1976.45.6.0709 978243

[7] Ellis ME, Smith CC, Reid TM, Porter IA. Chloramphenicol-resistant Salmonella typhimurium meningitis in an adult. Br Med J (Clin Res Ed). 1981;283:273. 10.1136/bmj.283.6286.273 PMC1506338 6788285

[8] Rodriguez RE, Valero V, Watanakunakorn C. Salmonella focal intracranial infections: review of the world literature (1884-1984) and report of an unusual case. Rev Infect Dis. 1986;8:31–41. 10.1093/clinids/8.1.31 3513285

[9] Sharma S, Raja A, Shivananda PG. Isolation of Salmonella typhi from brain tumor--a case report. Indian J Med Sci. 1986;40:233–5. 3817939

[10] Noguerado A, Cabanyes J, Vivancos J, Navarro E, Lopez F, Isasia T, et al. Abscess caused by Salmonella enteritidis within a glioblastoma multiforme. J Infect. 1987;15:61–3. 10.1016/s0163-4453(87)91476-9 2822812

[11] Bossi P, Mion G, Brinquin L, Bonsignour JP. [Postoperative brain abscess caused by Salmonella enteritidis]. Presse Médicale Paris Fr 1983. 1993;22:130. 8493217

[12] Shanley DJ, Holmes SM. Salmonella typhi abscess in a craniopharyngioma: CT and MRI. Neuroradiology. 1994;36:35–6. 10.1007/BF00599192 8107994

[13] Fiteni I, Ruiz FJ, Crusells MJ, Sanjoaquin I, Guillen G. [Salmonella enteritidis multifocal infection of the central nervous system. Efficacy of new cephalosporins]. Presse Médicale Paris Fr 1983. 1995;24:309–11. 7899392

[14] Sarria JC, Vidal AM, Kimbrough III RC. Salmonella enteritidis brain abscess: case report and review. Clin Neurol Neurosurg. 2000;102:236–9. 10.1016/s0303-8467(00)00099-8 11154812

[15] Kumari P, Kan VL. Salmonella typhimurium Brain Abscess: Postoperative Complication. Clin Infect Dis. 2000;30:621–2. 10.1086/313729 10722467

[16] Schröder J, Palkovic S, Kipp F, Wassmann H. Salmonella enteritidis causing brain abscess and coxitis following intracranial surgery. Acta Neurochir (Wien). 2003;145:919–21. 10.1007/s00701-003-0106-2 14577015

[17] Aissaoui Y, Azendour H, Balkhi H, Haimeur C, Atmani M. [Postoperative meningitis caused by an unusual etiological agent: Salmonella enteritidis]. Neurochirurgie. 2006;52:547–50. 10.1016/s0028-3770(06)71365-0 17203905

[18] Sait M, Rahmathulla G, Chen TL, Barnett GH. Rare case of intracranial Salmonella enteritidis abscess following glioblastoma resection: Case report and review of the literature. Surg Neurol Int [Internet]. 2011[cited 2015 Nov 29];2. Available from: http://www.ncbi.nlm.nih.gov/pmc/articles/PMC3205504/. 10.4103/2152-7806.86226 PMC3205504 22059142

[19] Kwambana-Adams B, Darboe S, Nabwera H, Foster-Nyarko E, Ikumapayi UN, Secka O, et al. Salmonella Infections in The Gambia, 2005-2015. Clin Infect Dis Off Publ Infect Dis Soc Am. 2015;61 Suppl 4:S354–362. 10.1093/cid/civ781 26449952

[20] Schaller BJ, Modo M, Buchfelder M. Molecular Imaging of Brain Tumors: A Bridge Between Clinical and Molecular Medicine? Mol Imaging Biol. 2007;9:60–71. 10.1007/s11307-006-0069-9 17203238

[21] Tewari A, Padma S, Sundaram PS. The diagnostic role of 18-fluorodeoxyglucocose-positron emission tomography/computed tomography in occult bacteremia searching underlying primary disease. Ann Indian Acad Neurol. 2012;15:336–8. 10.4103/0972-2327.104353 PMC3548383 23349610

[22] Buchwald DS, Blaser MJ. A Review of Human Salmonellosis: II. Duration of Excretion Following Infection with Nontyphi Salmonella. Rev Infect Dis. 1984;6:345–56. 10.1093/clinids/6.3.345 6377442

[23] Forbes NS. Engineering the perfect (bacterial) cancer therapy. Nat Rev Cancer. 2010;10:785–94. 10.1038/nrc2934 PMC3756932 20944664

